# Comparative transcriptome analysis provides novel insights into molecular response of salt-tolerant and sensitive polyembryonic mango genotypes to salinity stress at seedling stage

**DOI:** 10.3389/fpls.2023.1152485

**Published:** 2023-04-12

**Authors:** Nusrat Perveen, M. R. Dinesh, M. Sankaran, K. V. Ravishankar, Hara Gopal Krishnajee, Vageeshbabu S. Hanur, Saud Alamri, Mahipal Singh Kesawat, Mohammad Irfan

**Affiliations:** ^1^ Division of Fruit Crops, ICAR-Indian Institute of Horticultural Research, Hesaraghatta Lakepost, Bengaluru, Karnataka, India; ^2^ Division of Biotechnology, ICAR-Indian Institute of Horticultural Research, Hesaraghatta Lakepost, Bengaluru, Karnataka, India; ^3^ Department of Botany and Microbiology, College of Science, King Saud University, Riyadh, Saudi Arabia; ^4^ School of Biological Sciences, Seoul National University, Seoul, Republic of Korea; ^5^ Plant Biology Section, School of Integrative Plant Science, Cornell University, New York, NY, United States

**Keywords:** mango, transcriptome, salinity stress, RNA-Seq, polyembryony, Mylepelian, Turpentine, SOS pathway

## Abstract

**Introduction:**

Increased soil salinity in the recent years has adversely affected the productivity of mango globally. Extending the cultivation of mango in salt affected regions warrants the use of salinity tolerant/resistant rootstocks. However, the lack of sufficient genomic and transcriptomic information impedes comprehensive research at the molecular level.

**Method:**

We employed RNA sequencing-based transcriptome analysis to gain insight into molecular response to salt stress by using two polyembryonic mango genotypes with contrasting response to salt stress viz., salt tolerant Turpentine and salt susceptible Mylepelian.

**Results:**

RNA sequencing by Novaseq6000 resulted in a total of 2795088, 17535948, 7813704 and 5544894 clean reads in Mylepelian treated (MT), Mylepelian control (MC), Turpentine treated (TT) and Turpentine control (TC) respectively. In total, 7169 unigenes annotated against all the five public databases, including NR, NT, PFAM, KOG, Swissport, KEGG and GO. Further, maximum number of differentially expressed genes were found between MT and MC (2106) followed by MT vs TT (1158) and TT and TC (587). The differentially expressed genes under different treatment levels included transcription factors (bZIP, NAC, bHLH), genes involved in Calcium-dependent protein kinases (CDPKs), ABA biosynthesis, Photosynthesis etc. Expression of few of these genes was experimentally validated through quantitative real-time PCR (qRT-PCR) and contrasting expression pattern of Auxin Response Factor 2 (ARF2), Late Embryogenesis Abundant (LEA) and CDPK genes were observed between Turpentine and Mylepelian.

**Discussion:**

The results of this study will be useful in understanding the molecular mechanism underlying salt tolerance in mango which can serve as valuable baseline information to generate new targets in mango breeding for salt tolerance.

## 1 Introduction

Soil salinity is among the most deleterious abiotic stresses resulting in reduced crop productivity worldwide. Most mango cultivars have been reported to be highly sensitive to soil or water salinity particularly at the seedling stage resulting in poor seedling growth, scorching of leaf tips and margins and curling of leaves leading to plant death in severe cases (Bajpai et al., 2017; Deivasigamani et al., 2018). Hence, genetic improvement for salt tolerance and development of tolerant rootstocks could be helpful in improving the overall productivity of mango globally. However, salt-tolerance is a multi-genic trait with considerably low genetic variability in the available germplasm. This has limited the use of conventional breeding methods like hybridization and selection for developing salt tolerant rootstocks in mango. Understanding the molecular mechanism and bioprospecting major genes involved in salinity stress tolerance could facilitate efficient utilization of various genomic and genetic methods for improving salt stress tolerance in this commercially valuable fruit crop.

Salinity causes two major stresses in plants viz., osmotic stress, leading to reduced plant water uptake and ionic stress, caused mainly by toxic effects of sodium and chloride ions in plant cells (Munns and Tester, 2008). Maintenance of ion homeostasis and scavenging of free radicals by enzymatic (viz., superoxide dismutase, peroxidase and catalase) and non-enzymatic antioxidants are the major mechanisms involved in mitigating salt stress in plants (Slama et al., 2015; Yang and Guo, 2018). The salt overly sensitive (SOS) pathway comprising of three major components viz., SOS1, SOS2 and SOS3 is crucial for ionic stress signal transduction and is the foremost abiotic stress signalling pathway established in plants (Wang et al., 2023).

Transcriptome analysis helps in elucidating complexity of gene expression during different phases of plant growth and development under stress conditions. In the recent past, RNA-Seq has emerged as a method of choice for transcriptomic studies with high accuracy and sensitivity especially in crops where genome sequence information is lacking (Kogenaru et al., 2012; Rahman et al., 2014; Jazi et al., 2017). Recently this technique has been used for analysing transcriptomes of various crops under different stress conditions (Rahman et al., 2014; Jazi et al., 2017; Das and Majumder, 2019). Transcriptome analysis of salt tolerant *Malus* resource ZM-4 under salinity stress revealed higher concentration of flavonoids along with up-regulation of genes related to the flavonoid synthesis pathway indicating high antioxidant capacity of this salt tolerant resource (Wang et al., 2023). In citrus majority of genes induced by salt stress were involved in chloride ion homeostasis (*CCC1*), ion transport (*NHX1* and *HKT1*), biosynthesis of compatible osmolytes like proline (*P5CS*) and glycine betaine (*CMO*) and synthesis of enzymatic antioxidants (*APX*) (Snoussi et al., 2022).

In mango, transcriptomic studies have been done to identify defence related genes against *Colletotrichum gloeosporioides* in post-harvest mango fruits (Hong et al., 2016), to study ripening associated genes (Srivastava et al., 2016; Wu et al., 2022), floral malformation (Yadav et al., 2020) and identifying the genes involved in fruit development (Karim et al., 2022). However, as far as we are aware, the transcriptomic studies on mango under salt stress have notyet been undertaken. Hence, in the present study we selected two polyembryonic mango genotypes with contrasting response to salinity stress viz., salt tolerant Turpentine and salt susceptible Mylepelian (Nimbolkar, 2018) to elucidate the transcriptional variations among control and salt treated plants of these two genotypes by employing RNA-Seq method. Differential gene expression was studied and their functional annotation was carried out. Further, we also analysed the expression patterns of some key salinity stress-responsive genes and validated them using quantitative realtime PCR (qRT-PCR).

Being the first report on transcriptome analysis of mango under salinity stress, this studywill provide vast informative data besides facilitating functional genomics studies in mango. Furthermore, since different abiotic stresses like salinity, cold and drought have similar signal transduction pathways and plant response mechanisms, the generated information will also serve as a valuable resource for studying the expression of genes under other abiotic stresses and detecting stress-related genes required for management of various abiotic stresses in this major *Mangifera* species.

## 2 Materials and methods

### 2.1 Selection and collection of plant material

This experiment was carried out at ICAR-Indian Institute of Horticultural Research (IIHR), Bengaluru during the year 2019-2021. For transcriptome analysis, the kernels of two polyembryonic mango genotypes viz., Turpentine (salt-tolerant) and Mylepelian (salt-sensitive) (Nimbolkar, 2018) were collected from mango orchard of ICAR-IIHR, sown in polybags and transferred to shade net house where they were maintained for 1 year by following standard growing procedure. For salt treatment 5 plants each from salt-sensitive and salt-tolerant genotype were used with three biological replicates (total 15 plants for each genotype) at four time point viz., 0, 5, 10 and 15 days after salt treatment. These plants were then irrigated with 50mM NaCl solution at 2 days interval to impose salt stress and normal tap water was used to irrigate the control plants. The leaves of 15 plants were harvested separately at each time point, wiped thoroughly with 70% ethanol, flash frozen in liquid nitrogen after wrapping in properly labelled aluminium foil and brought to laboratory for extraction of RNA.

### 2.2 RNA extraction and library preparation

RNA isolation was carried out for both the genotypes separately at each time point after salt treatment (as discussed above) in three replications. Every time freshly harvested leaf samples were used for RNA extraction which was done using the extraction method given by Reddy et al. (2015). The quantity and purity of each RNA sample was determined using NanoDrop followed by quality check using 1.2% denatured agarose gel electrophoresis.

The RNA-Seq paired end sequencing libraries were prepared from the RNA samples which passed the quality control test using illuminaTruSeq Stranded mRNA sample Prep kit. Enrichment of mRNA from the total RNA was done using Poly-T attached magnetic beads, followed by enzymatic fragmentation, first strand cDNA conversion with the help of SuperScript II and Act-D mix to facilitate RNA dependent synthesis. The first strand cDNA was used for the synthesis of second strand by using the second strand mix. Finally, purification of dsc DNAwas done using AMPure XP beads followed by A-tailing, adapter ligation and further enrichment by limited number of PCR cycles.

### 2.3 Quantity and quality check (QC) of library

The PCR enriched libraries were analyzed for quantity and quality on Agilent 4200 TapeStation system using High Sensitivity D1000 ScreenTapeassay by following the manufacturer instructions and Qubit concentration and the mean peak sizes were obtained.

### 2.4 Cluster generation and sequencing

For cluster generation and sequencing, the PE illumina libraries were loaded onto Novaseq6000 for Paired-End sequencing that allows the template fragments to be sequenced in both the forward and reverse directions. The adapters were designed such as to allow selective cleavage of the forward strands after re-synthesis of the reverse strand during sequencing. The copied reverse strand was used to sequence from the opposite end of the fragment. The methods and software used in bioinformatics analysishave been presented in **Supplementary Table 1**.

### 2.5 Quantitative real-time PCR

Quantitative real-time PCR (qRT-PCR) was performed for the validation of selected transcripts related to salinity-responsive genes. OLIGO Primer Analysis Software v. 7 was used to design the required primers and Actin gene was utilized as an internal control for normalizing the gene expression level (Wen et al., 2019). The primers used in qRT-PCR analysis have been presented in **Supplementary Table 2**. QRT-PCR was performed by using SYBR Green real-time PCR master mix (Ampliqon, Denmark, Catalogue number, RR820A). The reaction mixture contained 2.0 μL of diluted cDNA sample as a template, 1 μL each of the forward and reverse primers (10 μM) and 5 μL real-time master mix making a final volume of 20 μL. The PCR cycling conditions have been presented in **Supplementary Table 3**. The 2^–ΔΔCt^ method was used to calculate the relative expression levels (Livak and Schmittgen, 2001).

## 3 Results

### 3.1 Mango transcriptome sequencing and transcriptome reconstruction

To gain an insight into the mechanism of salinity tolerance in mango at whole transcriptome level, transcriptomic changes in the seedlings of salt tolerant mango genotype Turpentine and salt susceptible mango genotype Mylepelian was investigated by RNA-Seq using the platform Novaseq6000. The transcriptome data was generated for four samples viz., Mylepelian seedlings treated with 50mM NaCl (MT), untreated or control Mylepelian seedlings (MC), Turpentine seedlings treated with 50mM NaCl (TT) and untreated or control Turpentine seedlings (TC). Here, for different samples, the total raw reads generated were 4154846, 19445963, 8419934 and 6433093 for MT, MC, TT and TC respectively (**Supplementary Table 4**). To remove low quality reads, data was filtered and a total of 2795088, 17535948, 7813704 and 5544894 clean reads were retained in MT, MC, TT and TC respectively for transcriptome assembly and further analysis. The percentage of G and C base numbers of total bases (GC%) was 49.86%, 47.31%, 46.59% and 47.42% for MT, MC, TT and TC respectively and more than 75.53% reads had Phred-like quality scores at Q30 (**Supplementary Table 4**).

Further, Trinity software was used for *de novo* assembly of clean reads in order to obtain assembly transcriptome and Hierarchical clustering was performed to remove redundance. The longest transcripts of each cluster were selected as unigenes. Length distribution information of transcripts and unigenes have been presented in the **Supplementary Tables 5**, **6**.

### 3.2 Gene functional annotation

For comprehensive gene functional annotation, the obtained unigenes were annotated against seven databases viz., NR (NCBI non-redundant protein sequences), NT (NCBI nucleotide sequences), PFAM(Protein family), KOG (euKaryotic Orthologous Groups), Swiss-Prot (protein sequence database), KEGG (Kyoto Encyclopedia of Genes and Genome) and GO (Gene Ontology). Out of total 41385 unigenes, 38139 (92.16%) were annotated in NR, 33015 (79.78%) were annotated in NT, 15531 (37.53%) were annotated in KOG, 31169 (75.31%) were annotated in Swissport, 29244 (70.66%) were annotated in PFAM, 25567 (61.78%) were annotated in GO and 11817 (28.55%) were successfully annotated in KOG. While 17.32% (7169 unigenes) were annotated in all the databases, 38811 (93.78%) unigenes could be annotated in at least one database (**Table 1**).

**Table 1:** The ratio of successfully annotated genes.

Statistical Items	Number of Unigenes	Percentage (%)
**Annotated in NR**	38139	92.16
**Annotated in NT**	33015	79.78
**Annotated in KO**	15531	37.53
**Annotated in Swissport**	31169	75.31
**Annotated in PFAM**	29244	70.66
**Annotated in GO**	25567	61.78
**Annotated in KOG**	11817	28.55
**Annotated in all Databases**	7169	17.32
**Annotated in at least one Database**	38811	93.78
**Total Unigenes**	41385	93.78

**Figure 1:**
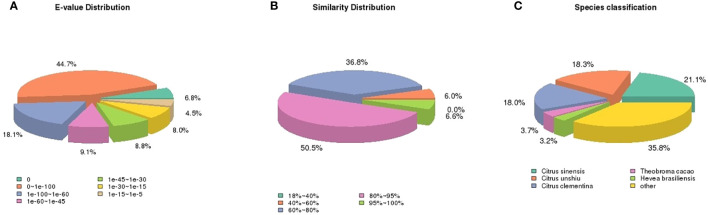
Pie charts showing distribution of the BLASTxmatches of mango transcriptome unigenes against Nr databases. **(A)** E-values distribution, **(B)** similarity distribution, and **(C)** species classification.

**Figure 2:**
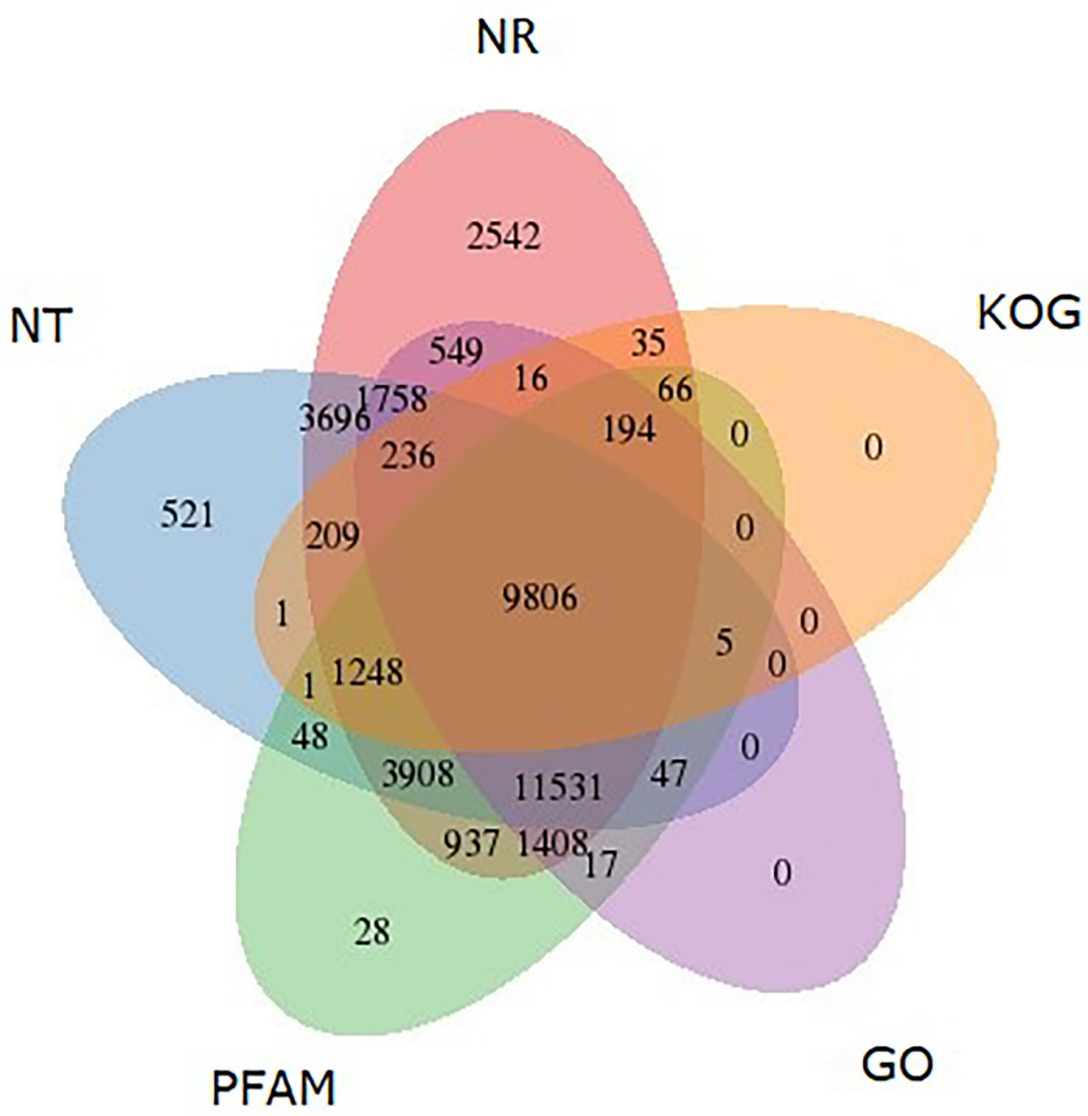
Venn diagram shows the BLAST results of *M. indica* against five databases, including NT, NR, KOG, GO and PFAM. The number of transcripts with significant hits is presented in each intersection of the Venn diagram.

Among the annotated unigenes, 87.5% were found to be significantly homologous (E-values<1e^-30^) to the sequences in NR database (**Figure 1A**) while 93.9% sequences had more than 60% similarity as indicated by similarity distribution (**Figure 1B**). Further, 21.1% of annotated sequences were found to have highest homology to sequences from *Citrus sinensis*, followed by *Citrus unshiu* (18.3%) and *Citrus clementina* (18%) (**Figure 1C**). After KOG-based annotation, 11817 annotated putative proteins were assigned to 26 functional categories of which the highest number of sequences was assigned to “post translational modification, protein turnover, chaperones” followed by “general function prediction only” and “translation, ribosomal structure and biogenesis”. In the metabolism group, “Lipid transport and metabolism”, “Carbohydrate transport and metabolism” and “amino acid transport and metabolism” were also highly represented. Altogether, 9806 sequences were found to be similar to proteins in NR, NT, PFAM, GO and KOG (**Figure 2**).

**Figure 3:**
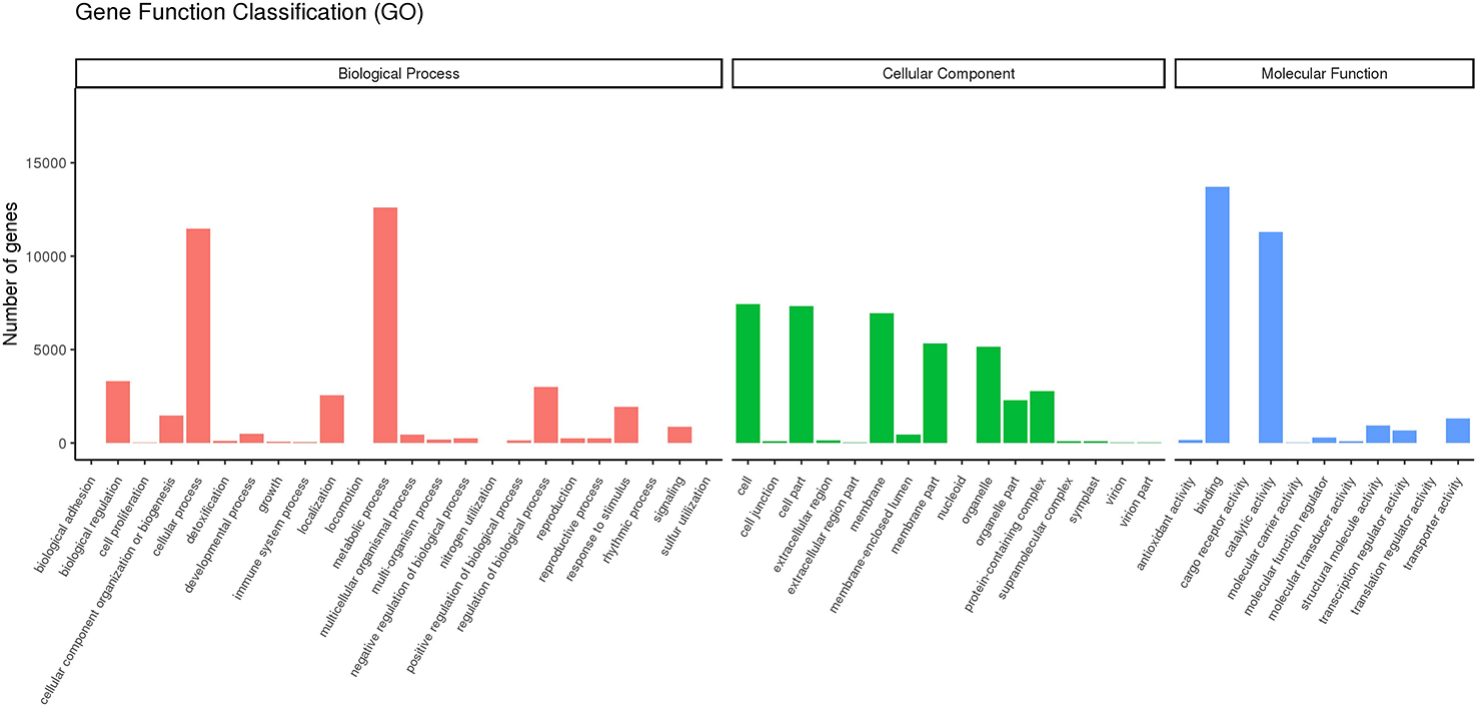
Gene Function Classification. Main GO categories Biological Process (BP), Cellular Component (CC), Molecular Function (MF).

Among the 25567 unigenesannotated with GO, most of the GO terms were assigned to biological process where genes involved in “metabolic process”, “cellular process” and “regulation of biological process” were highly represented while “binding”, “catalytic activity” and “transporter activity” & “cell”, “cell part” and “membrane” were the most enriched GO terms in the category molecular function and cellular component respectively (**Figure 3**).

**Figure 4:**
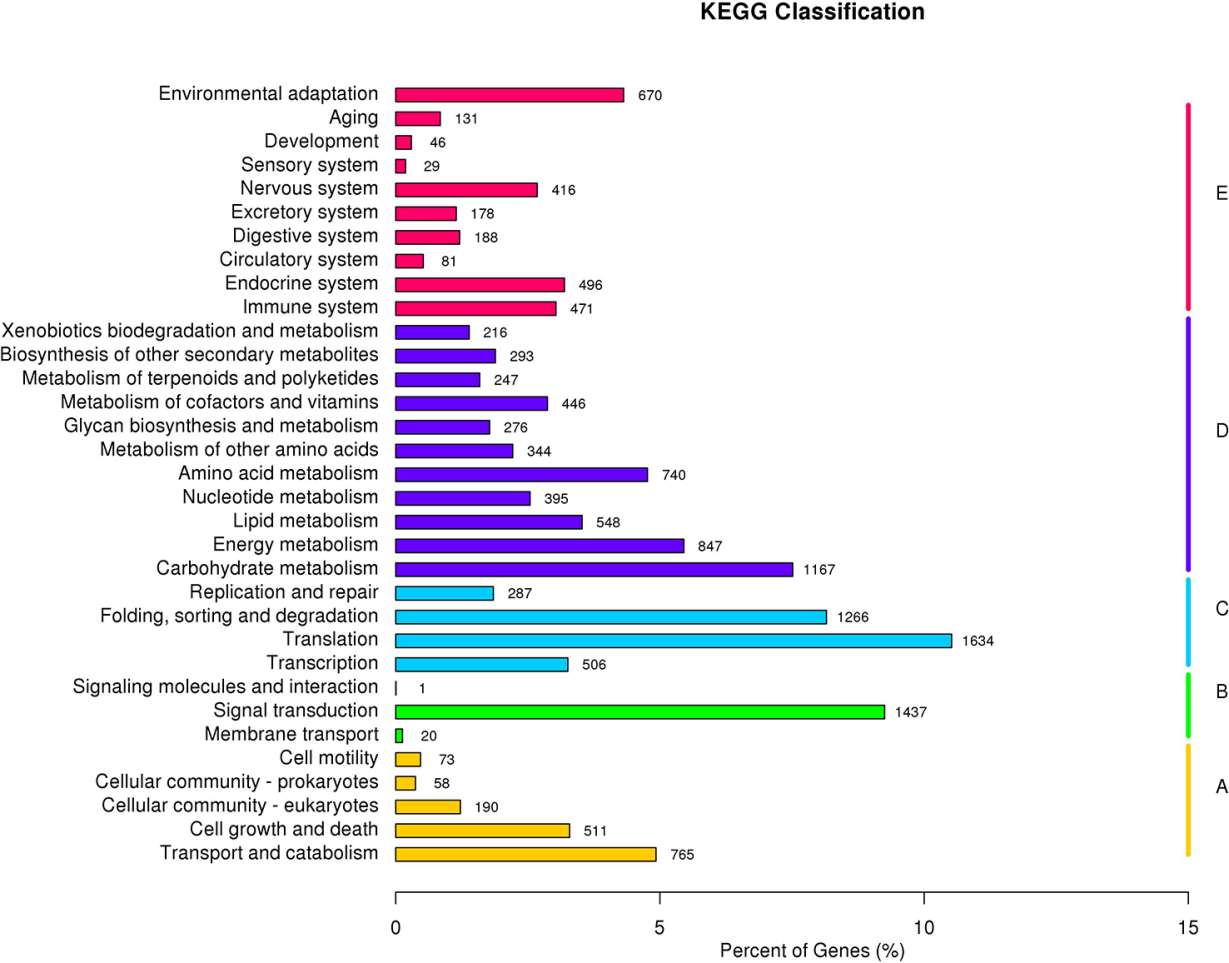
KEGG (Kyoto encyclopedia of genes and genomes) classification of Mango transcriptome.

Furthermore, for the identification of biological pathways in mango transcriptome, the potential protein sequences were searched against the KEGG database. Altogether 14973 unigenesgrouped into 33 major KEGG pathways covering five main KEGG categories viz., cellular processes, environmental information processing, genetic information processing, metabolism and organismal systems (**Figure 4**). Maximum number of sequences 5519 (36.85%) were involved in the KEGG category “metabolism” among which the most representative pathways were ‘carbohydrate metabolism’ involving 1167 (7.88%) unigenes followed by “energy metabolism” (847 unigenes) and “amino acid metabolism’ (740 unigenes).

### 3.3 Identification of transcription factors (TFs)

A homology search against plant TF database indicated abundance of four transcription factor families viz., C2H2, bZIP, NAC and bHLHin the mango transcriptome.

### 3.4 Differential gene expression profile of salt tolerant (Turpentine) and salt susceptible (Mylepelian) mango genotypes in response to salinity stress

**Figure 5:**
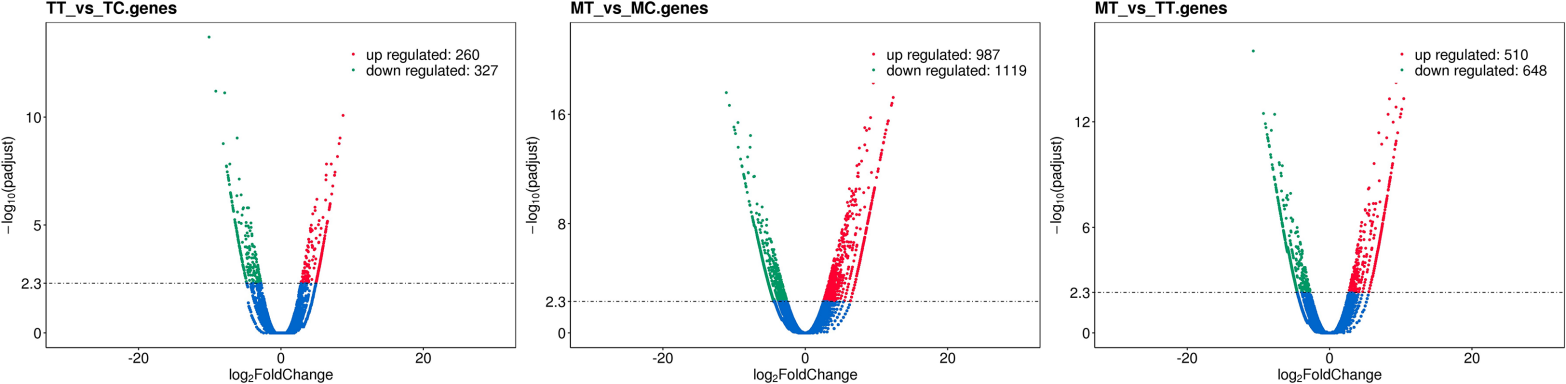
Volcano plots of DEGs in the pairwise comparisons and pathway enrichment. **(A)** Volcano plot of DEGs in the TT vs. TC comparison. Green dots represent genes upregulated in Turpentine Treated. Red dots represent genes upregulated in Turpentine Control. **(B)** Volcano plot of DEGs in the MT vs. MC comparison.Green dots represent genes upregulated in Mylepelian Treated. Red dots represent genes upregulated in Mylepelian Control. **(C)** Volcano plot of DEGs in the MT vs. TT comparison.Green dots represent genes upregulated in Mylepelian Treated. Red dots represent genes upregulated in Turpentine Treated. x-axis: fold change in gene expression between different samples; y-axis: statistical significance of the differences represented by red dots.

To specifically identify salt responsive differential gene expression in the two mango genotypes, we studied the expression profiles to understand the gene activity changes by comparing between, MT vs. MC, MT vs. TT and TT vs. TT using volcano plots to comprehend the distribution of differentially expressed genes (DEGs) (**Figures 5A–C**). DEGs with [log2(Fold Change)] > 1 and q-value < 0.005 were assumed to be significantly up or down-regulated in a sample in relation to the other one. Between the samples MT vs. MC, 2106 genes exhibited significant changes in gene expression where 987 genes were found to be up-regulated while 1119 genes were down-regulated (**Figure 5**). Among the top most up-regulated genes, we detected genes involved in abiotic stress tolerance viz., SKP1-like protein (*Arabidopsis thaliana*), transcription factors viz., NAC-domain containing protein (*A. thaliana*), bZIP transcription factor 60 (*A. thaliana*)and antioxidants viz., Glutathione S-transferase L3 (*A. thaliana*). While among the top most downregulated genes were the genes involved in photosynthesis and carbohydrate metabolism viz., Photosystem I reaction center subunit IV [*Thermosynechococcus elongatus* (strain BP-1)], Photosystem II 10 kDa polypeptide (*Nicotiana tabacum*).

The comparison between samples TT and TC revealed that a total of 587 genes had differential gene expression wherein 260 genes showed up-regulation while 327 genes were down-regulated (**Figure 5**). Among the top most upregulated genes in TT we found genes involved in abiotic stress tolerance viz., Late embryogenesis abundant protein D-34 (*Gossypium hirsutum)*, genes involved in regulation of transcription factor activity viz., Heterogeneous nuclear ribonucleoprotein 1 (*A. thaliana*) and transcription factors viz., NAC domain-containing protein 82 (*A. thaliana*). Among the major downregulated genes were the genes involved in photosynthesis and carbohydrate metabolism viz., Photosystem I reaction center subunit IV (*Synechococcus elongatus*), and Ribulose bisphosphate carboxylase small chain, (*Malus* sp.).

In comparison between MT vs TT, 510 were up-regulated and 648 genes were found to be down-regulated (**Figure 5**; **Table 2**). Among the topmost upregulated genes in TT were genes involved in carbon fixation and sucrose metabolism viz., Fructose-1,6-bisphosphate aldolase, (*A. thaliana*), genes responsible for maintaining lower cytoplasmic Na^+^ concentration and cellular pH homeostasis under salinity stress viz., vacuolar cation/proton exchanger 3, (*A. thaliana*), genes involved in antioxidant defence against abiotic stresses viz., Glutathione S-transferase F9 (*A. thaliana*), genes involved in compartmentalization of toxic Na+ ions in vacuoles during salt stress viz., V-type proton ATPase 16 kDa proteolipid subunit (*G.hirsutum*) and transcription factors involved in activating downstream stress-responsive genes viz., Probable WRKY transcription factor 19 (*A. thaliana*) while the top most downregulated genes in TT genes involved in protein degradation and turnover under different environmental stresses viz., SKP1-like protein 1B, (*A. thaliana*) and genes involved in maintaining cell integrity viz., Tol-Pal system protein TolB.

**Table 2:** Differentially expressed genes in tolerant Turpentine (TT) and susceptible Mylepelian (MT) mango genotypes after salt treatment.

Description	MT value	TT value	log2foldchange	Swissprot ID	Swissprot Description	Species
Carbohydrate and energy metabolism						
Cluster-5747.10930	0.488049	862.4153	-10.72	Q944G9	Fructose-bisphosphate aldolase 2, chloroplastic	*A. thaliana*
Cluster-11810.0	2.52656	207.2618	-6.3246	Q9M9R9	NADH dehydrogenase [ubiquinone] 1 beta subcomplex subunit 3-B	*A. thaliana*
Cluster-5747.11147	0.78955	334.1613	-8.65833	P0A423	Photosystem I reaction center subunit IV	*T. elongatus (strain BP-1)*
Cluster-5747.15107	12.81819	195.5565	-3.92731	P49107	Photosystem I reaction center subunit N, chloroplasti	*A. thaliana*
Cluster-5747.9860	4.189499	152.4327	-5.17574	P28475	NADP-dependent D-sorbitol-6-phosphate dehydrogenase	*Malus domestica*
Cluster-5747.10956	0.354492	37.29929	-6.65077	P60112	ATP synthase subunit 9, mitochondrial	*A. thaliana*
Cluster-5747.4204	0.462922	49.36202	-6.67	Q6Z1G7	Pyruvate dehydrogenase E1 component subunit beta-1, mitochondrial	*Oryza sativa subsp. japonica*
Cluster-5747.9055	0.519568	33.75501	-5.95557	Q1WIQ6	NADP-dependent glyceraldehyde-3-phosphate dehydrogenase	*A. thaliana*
Cluster-5747.4574	0.154463	8.217138	-5.66746	P29130	Phytochrome B	*N. tabacum*
Cluster-5747.11848	0.422724	22.78657	-5.68646	Q7X9A0	Ribulose bisphosphate carboxylase/oxygenase activase 1, chloroplastic	*Larrea tridentata*
Cluster-5747.7568	0.396619	19.69893	-5.56847	P37117	Cytochrome P450 71A4	*Solanum melongena*
Cluster-5747.13633	0.463484	22.91081	-5.56162	Q9LEI9	Enolase 2	*Hevea brasiliensis*
Cluster-5747.10858	62.36348	434.9252	-2.80137	P27493	Chlorophyll a-b binding protein 21, chloroplastic	*N. tabacum*
Cluster-6829.0	4.421023	42.53705	-3.2586	Q5HZ05	Probable trehalose-phosphate phosphatase J	*A. thaliana*
Cluster-5747.1916	0.624415	16.7558	-4.68143	H2DH21	Cytochrome P450 CYP72A219	*Panax ginseng*
Cluster-5747.17119	1.420603	45.4777	-4.93559	P46270	Cytochrome b-c1 complex subunit 9	*Solanum tuberosum*
Cluster-5747.10712	27.83108	237.6888	-3.09353	Q9SSV4	Inositol-3-phosphate synthase	*Nicotiana paniculata*
Cluster-5747.10333	0.273937	10.63951	-5.21408	Q09ME7	Cytochrome b6	*C. sinensis*
Cluster-5747.18045	2.764139	74.4993	-4.71964	O65194	Ribulose bisphosphate carboxylase small chain, chloroplastic	*Medicago sativa*
Cluster-5747.10932	0.873468	34.54165	-5.24004	Q40677	Fructose-bisphosphate aldolase, chloroplastic	*O. sativa* subsp. Japonica
Transcription related						
Cluster-5747.10612	0.522766	98.56596	-7.49201	O65683	bZIP transcription factor 11	*A. thaliana*
Cluster-5747.13295	0.291712	45.86876	-7.23013	Q9SZ67	Probable WRKY transcription factor 19	*A. thaliana*
Cluster-5747.11735	0.499532	51.03171	-6.60821	Q9FY93	NAC domain-containing protein 83	*A. thaliana*
Cluster-5747.10920	0.457929	144.3331	-8.23314	O82199	Zinc finger CCCH domain-containing protein 20	*A. thaliana*
Cluster-11830.1	0.471489	11.87525	-4.59018	Q9M9X4	Nuclear transcription factor Y subunit A-2	*A. thaliana*
Cluster-5747.15213	0.754476	124.8498	-7.30378	Q9LHJ8	Zinc finger A20 and AN1 domain-containing stress-associated protein 5	*A. thaliana*
Cluster-6931.0	3.177897	50.1189	-3.94743	Q9LNX4	Cyclin-dependent protein kinase inhibitor SMR5	*A. thaliana*
Cluster-5747.2860	0.410464	10.53146	-4.61685	Q9CA27	Ethylene-responsive transcription factor ERF118	*A. thaliana*
Cluster-5747.19505	0.382716	9.81952	-4.61685	Q9ZSI7	Probable WRKY transcription factor 47	*A. thaliana*
Cluster-5747.8961	0.418786	11.13929	-4.66875	Q9FY82	NAC domain-containing protein 82	*A. thaliana*
Cluster-5747.19765	0.543588	14.58684	-4.68143	Q9FNZ2	Zinc finger CCCH domain-containing protein 48	*A. thaliana*
Cluster-5747.12101	0.294863	8.051278	-4.70648	Q9SWF9	Zinc finger CCCH domain-containing protein ZFN-like	*Pisum sativum*
Cluster-5747.15153	0.489299	13.82108	-4.75531	Q9LXV5	Nuclear transcription factor Y subunit A-1	*A. thaliana*
Cluster-11633.1	0.286249	10.10699	-5.07675	O22208	bZIP transcription factor 17	*A. thaliana*
Cluster-5747.16002	0.278326	10.80999	-5.21408	Q93V43	Transcription factor TCP2	*A. thaliana*
Cluster-5747.6533	0.323712	14.0967	-5.37894	Q9ZUU0	WRKY transcription factor 44	*A. thaliana*
PROTEIN TRANSLATION, PROCESSING, AND DEGRADATION						
Cluster-5747.17910	0.557465	36.08586	-5.95033	P29344	30S ribosomal protein S1, chloroplastic	*Spinacia oleracea*
Cluster-5747.8191	0.95775	439.3906	-8.77465	Q9SGA6	40S ribosomal protein S19-1	*A. thaliana*
Cluster-5747.9427	2.142853	94.82812	-5.4455	Q9FJA6	40S ribosomal protein S3-3	*A. thaliana*
Cluster-5747.10116	3.576994	154.6448	-5.41187	P46297	40S ribosomal protein S23	*Fragaria ananassa*
Cluster-5747.9561	1.713642	115.7681	-6.01191	P49689	40S ribosomal protein S30	*A. thaliana*
Cluster-5747.11131	1.057101	59.96814	-5.76009	Q9UUC8	54S ribosomal protein L51, mitochondrial	*Schizosaccharomyces pombe (strain 972/ATCC 24843)*
Cluster-5747.7875	0.493087	92.27355	-7.48116	Q9LY66	50S ribosomal protein L1, chloroplastic	*A. thaliana*
Cluster-5747.16787	0.777502	39.34837	-5.59554	Q93Z17	50S ribosomal protein HLP, mitochondrial	*A. thaliana*
Cluster-5747.1927	0.855862	23.5709	-4.71884	Q02764	50S ribosomal protein L24, chloroplastic	*N. tabacum*
Cluster-5747.9945	2.171262	38.33191	-4.10993	P41127	60S ribosomal protein L13-1	*A. thaliana*
Cluster-5747.14774	0.858746	98.0378	-6.76843	P49690	60S ribosomal protein L23	*A. thaliana*
Cluster-5747.9311	2.156964	240.4931	-6.77833	P35130	Ubiquitin-conjugating enzyme E2 2	*M. sativa*
Cluster-5747.12649	0.404597	22.95234	-5.76009	Q9SYH3	E3 ubiquitin-protein ligase SPL2	*A. thaliana*
Cluster-5747.11034	0.618854	110.8561	-7.41812	O64937	Elongation factor 1-alpha	*O. sativa* subsp. japonica
Cluster-5747.14403	0.902876	78.10381	-6.40116	Q9FUM1	Elongation factor 1-gamma	*Prunus avium*
Cluster-5747.15515	0.433514	59.79806	-7.04124	Q9VHS8	Eukaryotic initiation factor 4A-III	*Drosophila melanogaster*
Cluster-5747.8577	0.41936	6.564413	-3.93664	Q03387	Eukaryotic translation initiation factor isoform 4G-1	*Triticum aestivum*
Cluster-5747.14632	1.551846	148.1243	-6.54308	Q96331	23.6 kDa heat shock protein, mitochondrial	*A. thaliana*
Cluster-5747.13416	7.076707	149.0875	-4.38878	P30236	22.0 kDa class IV heat shock protein	*Glycine max*
Cluster-5747.9037	0.831196	58.30507	-6.09882	P41152	Heat shock factor protein HSF30	*Solanum peruvianum*
Cluster-5747.5219	0.379297	9.642519	-4.60357	Q9SKY8	Heat shock 70 kDa protein 8	*S. peruvianum*
Cluster-5747.9058	10.97434	92.07416	-3.06435	Q9SYG1	17.4 kDa class III heat shock protein	*A. thaliana*
Cluster-5747.19474	0.444093	11.39429	-4.61685	Q2KI83	DnaJ homolog subfamily C member 17	*Bos taurus*
Cluster-5747.8031	0.437734	20.81368	-5.50564	Q8GYX8	Chaperone protein dnaJ 10	*A. thaliana*
Cluster-5747.10161	0.794474	40.58135	-5.60888	Q94JV4	Protein translation factor SUI1 homolog 2	*A. thaliana*
Cluster-5747.12495	14.23248	118.2612	-3.04869	Q9FLM8	DNA-directed RNA polymerases II, IV and V subunit 12	*A. thaliana*
Membrane and transport						
Cluster-5747.12805	0.459305	169.3091	-8.45904	Q93Z81	Vacuolar cation/proton exchanger 3	*A. thaliana*
Cluster-5747.8603	0.812204	179.1397	-7.7182	Q43434	V-type proton ATPase 16 kDa proteolipid subunit	*G. hirsutum*
Cluster-5747.9997	0.495966	68.87969	-7.05105	Q9HDY3	Membrane protein PB1A10.07c	*S. pombe (strain 972/ATCC 24843)*
Cluster-5747.7601	0.417414	11.10278	-4.66875	Q5XF36	Vacuole membrane protein KMS1	*A. thaliana*
Cluster-5747.19490	0.456562	15.26071	-4.99779	Q8L636	Sodium/calcium exchanger NCL	*A. thaliana*
Cluster-5747.14014	0.304374	11.60674	-5.18764	Q9SYG9	Cation/calcium exchanger 4	*A. thaliana*
Cluster-10132.0	1.550272	60.57633	-5.22278	Q9XGY4	Mitochondrial import inner membrane translocase subunit TIM8	*A. thaliana*
Signal transduction						
Cluster-5747.8287	0.236914	24.81632	-6.6443	Q9SCQ7	Acidic leucine-rich nuclear phosphoprotein 32-related protein	*A. thaliana*
Cluster-5747.13462	0.374466	14.36767	-5.1965	Q9M9C5	Probable leucine-rich repeat receptor-like protein kinase At1g68400	*A. thaliana*
Phyto-hormone Signaling Pathway						
Indole-3-acetic acid (IAA)						
Cluster-5747.11149	5.433301	1154.245	-7.71955	Q05349	Auxin-repressed 12.5 kDa protein	*F. ananassa*
Cluster-5747.5088	0.291823	7.143971	-4.54923	K4DF01	Auxin response factor 2B	*Solanum lycopersicum*
Cluster-5747.21031	0.44461	12.03549	-4.69401	Q38827	Auxin-responsive protein IAA9	*A. thaliana*
Ethylene (ETH)						
Cluster-5747.14043	0.263546	14.33032	-5.699	Q0WPQ2	Ethylene receptor 2	*A. thaliana*
Cluster-5747.4094	0.15246	3.911746	-4.61685	Q9S814	Ethylene-insensitive protein 2	*A. thaliana*
Abscisic acid (ABA)						
Cluster-5747.14365	1.353914	49.07933	-5.1469	Q65XK7	Protein phosphatase 2C 51	*O.sativa* subsp. japonica
Cluster-5747.6419	6.846892	68.59737	-3.32077	Q9LJK2	Abscisic acid 8’-hydroxylase 4	*A. thaliana*
Cluster-5747.1488	0.567819	19.51415	-5.03781	Q9S9Z7	Probable protein phosphatase 2C 10	*A. thaliana*
Cluster-5747.11300	0.449051	19.44913	-5.37113	P48578	Serine/threonine-protein phosphatase PP2A-4 catalytic subunit	*A. thaliana*
Cluster-5747.14365	1.353914	49.07933	-5.1469	Q65XK7	Protein phosphatase 2C 51	*O. sativa* subsp. japonica
Gibberellins (GA)						
Cluster-5747.10346	4.904498	84.94952	-4.10904	Q940G6	Gibberellin receptor GID1C	*A. thaliana*
STRESS AND DEFENSE						
Cluster-5747.9660	1.556587	463.1348	-8.18306	Q9ZQ80	Nodulin-related protein 1	*A. thaliana*
Cluster-5747.16139	0.480984	31.588	-5.97115	Q9T076	Early nodulin-like protein 2	*A. thaliana*
Cluster-5747.12383	0.735596	119.4745	-7.27686	O80852	Glutathione S-transferase F9	*A. thaliana*
Cluster-5747.11203	0.787111	25.93884	-4.97735	Q9LZ06	Glutathione S-transferase L3	*A. thaliana*
Cluster-5747.8181	0.843581	59.17386	-6.06613	P14831	Superoxide dismutase [Cu-Zn], chloroplastic	*S.lycopersicum*
Cluster-5747.10916	0.387568	23.81088	-5.87501	P49317	Catalase isozyme 3	*Nicotiana plumbaginifolia*
Cluster-5747.3380	0.903409	51.24937	-5.76009	P48534	L-ascorbate peroxidase, cytosolic	*P. sativum*
Cluster-5747.11416	0.756717	24.22475	-4.93559	Q42564	L-ascorbate peroxidase 3	*A. thaliana*
Cluster-5747.8136	0.618353	20.95974	-5.01794	Q9ZSK1	Tocopherol O-methyltransferase, chloroplastic	*A. thaliana*
Cluster-5747.6755	1.102863	35.82512	-4.95662	Q9M7I9	Stress enhanced protein 1, chloroplastic	*A. thaliana*
Cluster-5747.14351	1.20265	29.15839	-4.56708	Q6PL11	SKP1-like protein 1	*O. sativa* subsp. japonica
Cluster-5747.21026	0.495003	17.47776	-5.07675	Q42546	SAL1 phosphatase	*A. thaliana*
Cluster-5747.16012	0.846384	24.30602	-4.8111	Q84P54	Gamma aminobutyrate transaminase 1, mitochondrial	*S. lycopersicum*
Cluster-6633.0	6.613999	113.3052	-4.09138	P09444	Late embryogenesis abundant protein D-34	*G. hirsutum*
CELL WALL AND CYTOSKELETON METABOLISM						
Cluster-5747.13506	0.915311	114.8372	-6.90452	Q8SAG3	Actin-depolymerizing factor	*Vitis vinifera*
Cluster-5747.8757	1.974895	94.13603	-5.54166	P53492	Actin-7	*A. thaliana*
Cluster-10197.2	0.700536	20.77723	-4.82558	Q9M9G7	Probable F-actin-capping protein subunit beta	*A. thaliana*
Cluster-5747.11832	0.54013	29.75098	-5.7176	Q944S2	WD repeat-containing protein GTS1	*A. thaliana*
CALCIUM SIGNALING PATHWAY						
Cluster-5747.12071	0.252656	14.74919	-5.80136	A0A1P8BH59	Calmodulin binding protein PICBP	*A. thaliana*
Cluster-10300.0	1.544009	30.52923	-4.27322	Q93Z27	Probable calcium-binding protein CML46	*A. thaliana*
Cluster-5747.3393	0.341503	13.34412	-5.22278	C0SVV6	Calmodulin-binding protein 60 A	*A. thaliana*
Cluster-5747.15006	0.778294	30.96115	-5.24859	P30188	Probable calcium-binding protein CML35	*A. thaliana*
Cluster-6903.0	0.584762	25.8776	-5.40213	Q7FRS8	Calcineurin B-like protein 10	*A. thaliana*
PROTEIN KINASES						
Cluster-5747.13164	0.188016	4.779748	-4.60357	Q9C9U5	Probable serine/threonine-protein kinase SIS8	*A. thaliana*
Cluster-5747.7596	0.729975	19.24479	-4.65594	Q8GYA4	Cysteine-rich receptor-like protein kinase 10	*A. thaliana*
Cluster-11898.1	0.343344	9.051794	-4.65594	F4JTP5	Serine/threonine-protein kinase STY46	*A. thaliana*
Cluster-5747.14884	0.227533	8.462309	-5.15161	Q852L0	Casein kinase 1-like protein HD16	*O. sativa* subsp. Japonica
Cluster-5747.15663	5.513705	50.517	-3.19098	Q9ZQ31	Serine/threonine-protein kinase STY13	*A. thaliana*
Cluster-5747.15701	18.65338	199.485	-3.41709	A2YNT8	Serine/threonine-protein kinase SAPK2	*O. sativa* subsp. japonica
Cluster-5747.8252	0.484952	19.29178	-5.24859	P0C5D6	Serine/threonine-protein kinase SAPK3	*O. sativa* subsp. japonica

### 3.5 GO enrichment analysis for DEGs

DEGs set in all the studied samples were investigated by GO enrichment analysis and GO terms with padj< 0.05 were considered to be significant enrichment. Between the samples, MT vs. MC, ‘generation of precursor metabolites and energy’ and ‘photosynthesis’ were the most enriched GO terms in the category “biological process” with 68 and 36 differentially expressed genes respectively while ‘oxidoreductase activity’ (203 DEGs) and ‘mitochondrion’ (61 DEGs) were the most enriched GO terms in the category “molecular function” and “cellular components” respectively. Between the samples, MT vs. TT, ‘generation of precursor metabolites and energy’ was most enriched GO term in the category “biological process” with 32 differentially expressed genes while ‘oxidoreductase activity’ (112 DEGs) was most enriched GO term in the category “molecular function”. In the category “cellular component”, ‘cytoplasm’ (185 DEGs) and ‘ribosome’ (45 DEGs), ‘mitochondrion’ (35 DEGs) and ‘organelle’ (207 DEGs) were the most enriched GO terms. Further, between the samples, TT vs. TC, ‘photosynthesis’ (26 DEGs) and ‘generation of precursor metabolites and energy’ (25 DEGs) were the most enriched GO terms in the category “biological process” while ‘plastid’ (26 DEGs) and ‘thylakoid’ (25 DEGs) were most enriched GO terms in the category “cellular component” and trans-membrane transporter activity (38 DEGs) was the most enriched GO term in the category “molecular function”.

### 3.6 KEGG pathway enrichment analysis

Between the samples, MT vs. MC, the top most significantly DEGs enriched pathways (q-value < 0.05) were ‘oxidative phosphorylation’ (49 DEGs), ‘parkinson’s disease’ (37 DEGs), ‘cardiac muscle contraction’ (14 DEGs) and ‘photosynthesis’ (22 DEGs). Between the samples MT vs. TT, the most enriched KEGG pathways were ‘Amyotrophic lateral sclerosis (ALS)’ (10 DEGs), ‘oxidative phosphorylation’ (27 DEGs), ‘parkinson’s disease’ (22 DEGs) and ‘huntington’s disease’ (24 DEGs). While among the samples, TT vs. TC, ‘photosynthesis’ (16 DEGs), ‘galactose metabolism’ (8 DEGs), ‘cardiac muscle contraction’ (6 DEGs), ‘nitrogen metabolism’ (6 DEGs) and ‘oxidative phosphorylation’ (12 DEGs) were the most enriched KEGG pathways.

### 3.7 qRT-PCR analysis

**Figure 6:**
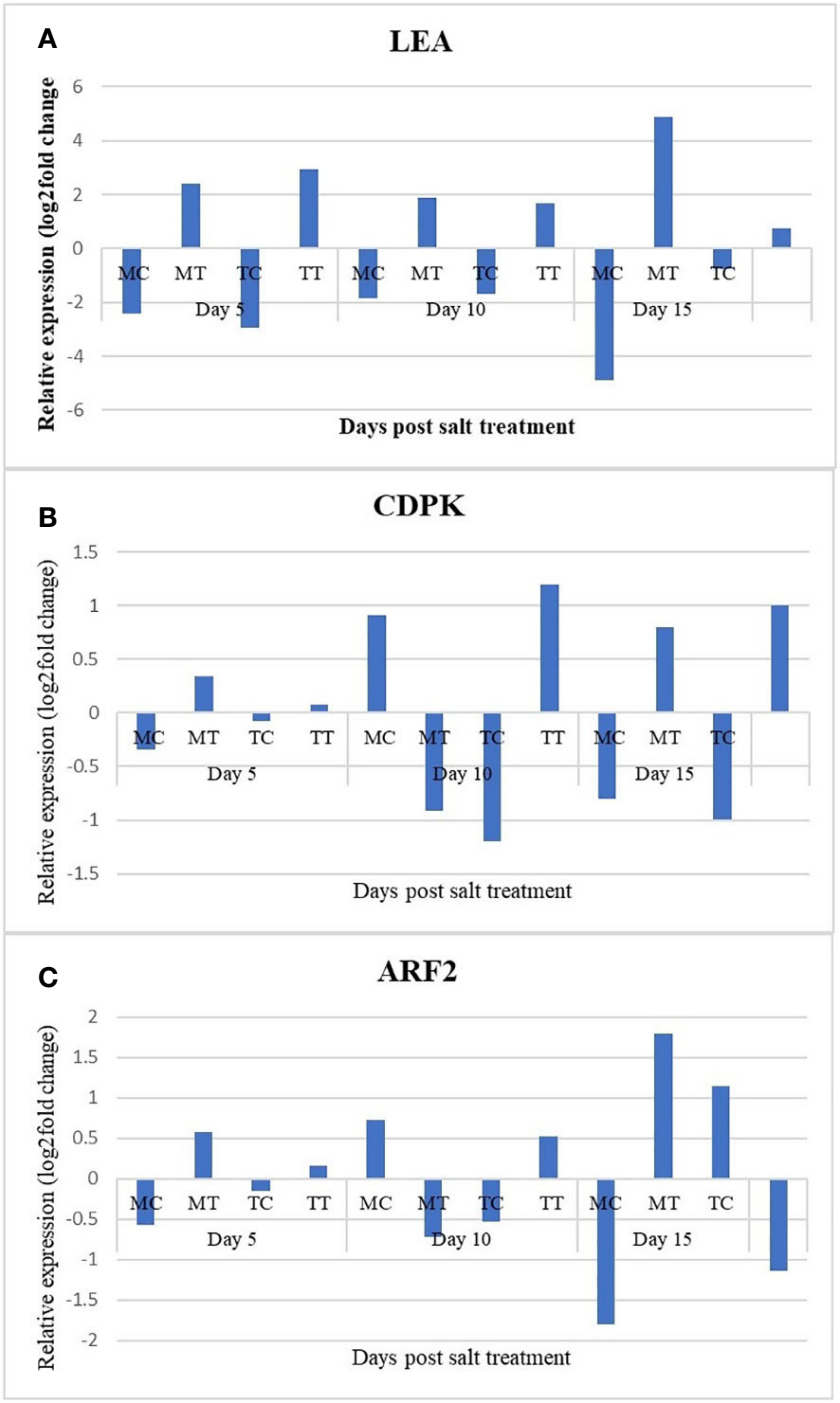
**(A–C)** Expression profiles of LEA, CDPK and ARF2 over different time points of salt stress (NaCl at the rate of 50mM) in leaves of mango genotypes Turpentine and Mylepelian in three comparisons. Mylepelian Treated (MT) vs. Mylepelian Control (MC); Mylepelian Treated (MT) vs. Turpentine Treated (TT) and Turpentine Control (TC) vs. Turpentine Treated (TT).x-axis: Days post salt treatment; y axis: Relative expression (log2fold change.Relative gene expression was calculated using 2−ΔΔCT method (fold change). * Significant differences for p-values < 0.05.

In order to validate the RNA-Seq data, qRT PCR analysis was done on the RNA samples initially used for transcriptome analysis using Late embryogenesis abundant (LEA) Auxin response factor 2 (ARF2), and Calcium-dependent protein kinase (CDPK), (**Figures 6A–C**). The relative expression level obtained was transformed to log_2_ Fold Change to compare between RNA-Seq and qRT-PCR expression data. Correlation analysis between the two data sets showed a strong positive correlation (R^2^ = 0.7939) indicating the accurateness of the RNA-Seq expression data in the present study. Furthermore, based on our qRT-PCR results, the expression of LEA was downregulated in MC up to 15 days of salt treatment as compared to MT and was upregulated in TT up to 15 days of salt treatment as compared to TC. With respect to the samples MT vs. TT, although the expression of LEA was upregulated in both samples up to 15 days of salt stress, the expression was found to be more in TT (+2.933 fold) as compared to MT (+2.404) at fifth day after salinity treatment it was significantly lower in TT (+0.757 fold) in comparison to MT (4.884) at 15^th^ day of salinity treatment. The expression of CDPK was downregulated in MC as compared to MT after 5^th^ day of salt treatment while it was slightly upregulated in TT as compared to TC at this time point. Moreover, in TT the highest expression for this gene as compared to TC was observed at 10^th^ day after salt treatment (+1.198-fold) with slight decline (+0.998-fold) at 15^th^ day after salt treatment. With respect to MT vs. TT, CDPK was found to be downregulated in MT (-0.909-fold) and upregulated in TT (+1.198-fold) which further changed at 15^th^ day of salt stress where CDPK was found to be upregulated in both the samples with higher level of expression in TT (+0.998-fold) as compared to MT (+0.803-fold). A decrease in the expression of ARF2 was observed at 15^th^ day after imposition of salt stress in all the three samples with highest downregulation in TT (-2.355-fold) as compared to TC. At 5^th^ day of salinity treatment, ARF2 was observed to be upregulated in both MT and TT plants and the expression was higher in MT (+0.575-fold) as compared to TT (+0.157-fold). While, at 10^th^ day of salt stress treatment, an opposite trend was observed wherein, ARF2 was found to be downregulated in MT (-0.718-fold) as compared to TT (+0.528-fold) which changed again at 15^th^ day of salt stress imposition and it was observed to be upregulated in MT (+1.798-fold) as compared to TT (-1.138-fold) where it was downregulated.

## 4 Discussion

Salinity results in oxidative, osmotic and ionic stress which in turn affects the growth and development of plants (Hussain et al., 2018). Elucidating the molecular mechanism of salinity tolerance in mango could facilitate efficient utilization of various genetic and genomic techniques for improving salinity tolerance in this commercially valuable fruit crop. Transcriptome analysis helps in understanding the complexity of gene expression during different growth and development phases as well as under stress conditions. RNA-Seq has emerged as a method of choice in the recent past for transcriptomic studies in crops lacking genome sequence information (Rahman et al., 2014; Jazi et al., 2017). Here, a total of 4154846, 19445963, 8419934 and 6433093 raw reads were generated for MT, MC, TT and TC respectively and after trimming of low-quality bases and adapters a total of 2795088, 17535948, 7813704 and 5544894 clean reads were retained in MT, MC, TT and TC respectively which were then *de novo* assembled to get assembly transcriptome using Trinity software (**Supplementary Table 4**). Trinity uses most of the sequencing reads for the construction of assembly besides producing comparatively a smaller number of fragmented transcript (Jazi et al., 2017).

Further, to understand the functions of assembled mango transcripts, they were annotated against sequence-based (NR, NT and Swiss-Prot) and domain-based (PFAM) databases (**Table 1**). Altogether, 9806 sequences were found to be similar to proteins in NR, NT, PFAM, GO and KOG (**Figure 2**). With respect to species distribution most of the annotated sequences exhibited homology to *C. sinensis* followed by *C.unshiu* and *C. clementina* (**Figure 1C**). Transcriptome analysis of pistachio, another member of family Anacardiaceae to which mango belongs, also revealed highest homology of the annotated sequences to *C. sinensis* (39.7%) and *C.clementina* (18%). *Mangifera indica* and *C. sinensis* have previously been reported as phylogenetically related species (Azim et al., 2014).

To predict the putative function of translated assembly we aligned them to the KOG database where 5-6% protein sequences fell under the category “function unknown”, indicating mango to be a phylogenetically distant species in comparison to those available in the KOG database. Similar reports are available in pistachio (Jazi et al., 2017). Further, GO studies suggested that the majority of annotated sequences are involved in regulating basic biological and metabolic processes (**Figure 3**). This is in congruence with the previous findings of highly representative GO terms in leaf and fruit transcriptome of mango and leaf, shoot and root transcriptome of pistachio (Dautt-Castro et al., 2015; Jazi et al., 2017). To specifically identify salt responsive differential gene expression in the two mango genotypes, we studied the expression profiles to understand the gene activity changes by comparing between, MT vs. MC, MT vs. TT and TT vs. TT using volcano plots to comprehend the distribution of differentially expressed genes (DEGs) (**Figures 5A–C**).

Transcription factors convert the stress-induced signals produced under abiotic stresses to plant cellular responses (Vom Endt et al., 2002; Wang et al., 2016). In the present study, several transcription factors exhibited differential expression under salinity stress among which four transcription factor families, C2H2, bZIP, NAC and bHLH were found to be most abundant (**Table 2**). TF families viz., bHLH, MYB, C2H2, bZIP, NAC, ERF, WRKY, and C3H were found to be differentially expressed in *Xanthoceras sorbifolia* seedlings under salt and saline-alkali stress (Wang et al., 2020). TF families such as bHLH, NAC, ERF, MYB, and WRKY have been found to be involved in response of plants to abiotic stress (Aprile et al., 2018; Shinde et al., 2019). Since specific transcription factors regulate activation of most of the stress-responsive genes, detection of major transcription factors will facilitate the understanding of molecular mechanisms of abiotic stress tolerance in mango.

### 4.1 Transcripts involved in synthesis of phyto-hormones involved in abiotic stress tolerance

Plants response to salt stress is very complicated process involving carbohydrate metabolism, secondary metabolite biosynthesis, biosynthesis of phyto-hormones and signal transduction pathways (Wang and Irving, 2011).

Phyto-hormones act as signalling molecules and trigger specific signalling cascades after sensing abiotic stress that brings about changes to help plants cope the adverse conditions. In the present study, the mango transcript assembly was found to capture many vital components of signal transduction pathways of major phyto-hormones like auxin, gibberellin and abscisic acid (ABA). Among the phytohormones, ABA is known to be the central regulator of abiotic stress tolerance in plants. KEGG analysis discovered ABA biosynthesis gene (9-cis-epoxycarotenoid dioxygenase), transcripts of enzyme involved in ABA catabolism (ABA 8’-hydroxylase) and ABA signalling components (Protein phosphatase 2C 51, Probable protein phosphatase 2C 10 and Serine/theonine-protein phosphatase PP2A-4 catalytic subunit) (**Figure 4**; **Table 2**). Jazi et al. (2017) detected genes involved in ABA biosynthesis pathway,enzymes involved in ABA catabolism (ABA 8’-hydroxyla), ABA signalling components in pistachio transcriptome assembly in response to salt stress. We also detected genes associated with auxin signal transduction pathway viz., auxin repressed 12.5 kDa protein, auxin response factor 2B and auxin responsive protein IAA9. Similar results have also been reported in pistachio under salt stress (Jazi et al., 2017). In addition to this, the transcripts related to ethylene signal transduction viz., ethylene receptor 2 and ethylene-insensitive protein 2 and gibberellin receptor GID1C was up-regulated in Turpentine while it was down-regulated in Mylepelian,

### 4.2 Transcripts related to carbohydrate metabolism

Several genes associated with carbohydrate and energy metabolism were found to be altered by salt stress in mango. In the present study, fructose-bisphosphate aldolase 2 and fructose-bisphosphate aldolase, key enzymes in the glycolytic pathway, were upregulated in Turpentine while it was downregulated in salt susceptible variety Mylepelian. Overexpression of fructose-bisphosphate aldolase (FBPA) has been reported to increase salt tolerance in tobacco by improving proline content (Konishi et al., 2005). Up-regulation of FBP3 aldolase is known to improve cotton root growth under salt stress by increasing levels of starch and sugars (Li et al., 2015). Cytochrome 450s belonging to the oxidoreductases class of enzymes play a major role in the biosynthesis of different metabolites and are also implicated in increasing abiotic stress tolerance in plants by increasing the activities of anti-oxidant compounds like flavonoids (Yan et al., 2016). In our study, we found Cytochrome P450 71A4, Cytochrome P450 CYP72A219 to be upregulated in salt treated Turpentine along with other transcripts like Photosystem I reaction center subunit IV, Cytochrome b-c1 complex subunit 9 and Cytochrome b6 (**Table 2**). Endoplasmic reticulum–localized cytochrome b5 in rice (*OsCYB5-2*)has been found to play a vital role in maintaining intracellular K^+^/Na^+^ homeostasis in rice cells and improve salinity tolerance in rice seedlings (Song et al., 2021).

### 4.3 Transcripts involved in transcription

Transcription factors play a vital role in regulating the expression of specific genes in response to various biotic and abiotic stresses in plants (Jiang et al., 2007). One of the largest transcription factor families in plants, bZIP (basic leucine zipper) protein, is the major regulator of ABA mediated abiotic stress signalling pathways in plants (Liang et al., 2016). Over-expression of *GhABF2*, encoding a typical cotton bZIPTF resulted in significant increase in drought and salt stress tolerance in Arabidopsis and cotton (Liang et al., 2016). In the present study we detected the upregulation of bZIP transcription factor 11 and bZIP transcription factor 17 in salt treated Turpentine. The other transcription factors upregulated in salt treated tolerant mango genotype Turpentine were probable WRKY transcription factor 19 and WRKY transcription factor 44. *MfWRKY70* of *Myrothamnus flabellifolia* was found to maintain ROS homeostasis and membrane stability under stress condition by enhancing the anti-oxidant enzyme system along with acting as a positive regulator of stress responsive genes like NCED3, RD29A and P5CS (Xiang et al., 2021). Moreover, we also detected the abundance of zinc finger transcription factor, Zinc finger CCCH domain-containing protein 20, Zinc finger A20 and AN1 domain-containing stress-associated protein 5 in Turpentine (**Table 2**). Zinc finger proteins have largely been documented in regulating stress related responses in plants and an over-expression of CCCH-type zinc finger proteins AtSZF1 and AtSZF2 is known to enhance the salinity tolerance in Arabidopsis (Sun et al., 2007).

### 4.4 Transcripts related to metabolism of proteins

Protein turnover defined as the balance between protein synthesis and degradation is highly significant to attain a cumulative cellular response (Reinbothe et al., 2010). Various transcripts involved in translation, processing and degradation of proteins were observed in our study. Several ribosomal proteins including 30S ribosomal protein S1, 40S ribosomal protein S19-1, 60S ribosomal protein L13-1 among others were upregulated in salt tolerant mango genotype Turpentine. Earlier reports suggest that ribosomal proteins may increase or decrease under salt stress (Rodriguez-Uribe et al., 2011; Li et al., 2015). Moreover, our study showed increased concentration of Eukaryotic initiation factor 4A-III, Eukaryotic translation initiation factor isoform 4G-1 and Elongation factor 1-alpha in salt treated Turpentine as compared to Mylepelian (**Table 2**). Li et al. (2015) also reported higher abundance of elongation factor gi|6015064 under salt stress conditions in cotton. Among different families of eukaryotic initiation factors (eIFs), the role of eIF1A has been demonstrated in abiotic stress tolerance (Singh et al., 2007; Wang et al., 2012). Li et al. (2019) identified 18 eIF genes from mango transcriptome and found that majority of the *MieIF* genes were involved with abiotic stress in mango among which the expression of*MieIF1A-a*, significantly increased under salinity stress.The upregulation various translation related transcripts in Turpentine indicates the involvement of synthesis of stress related proteins to help it cope with salinity stress. Hsp70 plays an important role in appropriate folding of polypeptides and translocation of precursor proteins (Li et al., 2015). Different members of heat shock proteins viz., 22.0 kDa class IV heat shock protein; 23.6 kDa heat shock protein, Heat shock factor protein HSF30, Heat shock 70 kDa protein 8 and 17.4 kDa class III heat shock protein along with Chaperone protein dnaJ were found to be upregulated in Turpentine.

### 4.5 Transcripts related to cell wall and cytoskeleton metabolism

Adjustment of cell size by remodelling of cytoskeleton is highly significant for maintaining the turgor pressure of cell under salinity stress (Zhang et al., 2012). In salt treated Turpentine leaves, two actin-binding proteins Actin-depolymerizing factor and Actin-7 were found to be upregulated. Previously, three actin-binding proteins viz., actin depolymerizing factor, actin related protein-2 and actin-binding protein 29 were reported to affect cell wall and cytoskeleton remodelling in salt treated roots of cotton (Li et al., 2015). Hence, it can be suggested that depolymerization and reorganization of actin cytoskeleton is implicated in improving salt tolerance in Turpentine.

### 4.6 Transcripts related to membrane and transport

Major damage caused by salinity stress to plants is attributed to the hyper-osmotic stress and ionic toxicity owing to the increase in the concentration of Na^+^ and Na^+^/K^+^ ratio. The vacuolar type H^+^ -ATPase plays a significant role in sequestering sodium ion into the central vacuole, thus, maintaining the ion homeostasis (Golldack and Dietz, 2001). In our study, transcripts related to different membrane and transport proteins such as Vacuolar cation/proton exchanger 3, V-type proton ATPase 16 kDa, Sodium/calcium exchanger NCL, Cation/calcium exchanger 4 were found to be upregulated under salt treated conditions in Turpentine (**Table 2**). The cation/calcium exchangers are calcium transporters playing significant role in calcium signalling pathways (Cai and Lytton, 2004). The upregulation of membrane proteins suggests their potential role in maintaining the ion-homeostasis in salt tolerant mango genotype Turpentine thus mitigating the ionic toxicity part of salinity stress and may play an important role in salt-stressed responses.

### 4.7 Transcripts related to signal transduction

Leucine-rich repeat receptor-like kinases (LRR-RLKs), are involved in various signal transduction pathways associated with hormone and abiotic stress responses (Ten Hove et al., 2011). In rice a novel LRR-RLK gene *OsSTLK*, has been reported to confer salinity tolerance by regulating ROS scavenging along with stomatal patterning (Lin et al., 2020). In the present study the up-regulation of Probable leucine-rich repeat receptor-like protein kinase At1g68400 suggests its function in plant and Na^+^ interaction, recognition and signal transduction improving salt stress tolerance in mango genotype Turpentine.

### 4.8 Transcripts related to calcium signalling pathway and osmotic adjustment

Osmotic stress during initial stage of salinity stress is the result of increased osmotic potential of the rhizosphere owing to the accumulation of Na^+^ and Cl^-^ ions. Many tolerance strategies adopted by plants to cope with salt stress such as accumulation of osmoprotectants, antioxidant machinery are also coordinated by Ca^2+^ signalling (Seifikalhor et al., 2019). In our study, several transcripts related to calcium signalling pathways were found to be upregulated in the leaves of salt treated Turpentine such as Probable calcium-binding protein CML35 and Probable calcium-binding protein CML46 (**Table 2**). Further, the initial Ca^2+^-dependent signalling network has been postulated to be involved in the salt stress responses consisting Ca^2+^ transport and downstream targets like calmodulin (CaM, a calcium modulated protein), CaM-like proteins, calcineurin-B-like protein (CBLs) and CBL-interacting protein kinases (Wan et al., 2018). In our study, Calmodulin binding protein PICBP, Calmodulin-binding protein 60 A and Calcineurin B-like protein 10 were found to be downregulated in salt susceptible genotype Mylepelian while it was upregulated in the salt tolerant mango genotype Turpentine suggesting that robust calcium sensing and signalling pathways operating in Turpentine could be implicated in its ability to cope with salt stress.

### 4.9 Transcripts related to protein kinases

Maintaining K^+^/Na^+^ ratio is critical for plant cells to survive under salt stress. Protein Kinases are shown to be vital for maintenance of intracellular Na^+^ and K^+^ homeostasis and salt tolerance in plants (Liu et al., 2000). In the present study, transcripts related to protein kinases such as Serine/threonine-protein kinase STY46, Serine/threonine-protein kinase SAPK2 and Serine/threonine-protein kinase SAPK3 were found to be upregulated in salt treated Turpentine. The abundance of serine-threonine protein kinases in Turpentine suggests the involvement of SOS2 protein kinase activity for imparting salt tolerance to this polyembryonic mango genotype (**Table 2**). SOS pathway comprising SOS1 (a plasma membrane Na^+^/H^+^ antiporter), SOS2 (a serine/threonine protein kinase) and SOS3 (Calcium binding protein) has been reported to be involved in conferring salt tolerance in pistachio (Jazi et al., 2017).

### 4.10 Transcripts related to stress and defence

Taking into account the significance of antioxidants in alleviating salinity stress, we surveyed the assembled mango transcriptome for stress responsive transcripts encoding anti-oxidants. Transcripts related to several enzymatic anti-oxidants such as Glutathione S-transferase F9, Superoxide dismutase [Cu-Zn], Catalase isozyme 3, L-ascorbate peroxidase 3 and Tocopherol O-methyltransferase were found to be up-regulated in salt-treated Turpentine as compared to Mylepelian. Abundance of transcripts for superoxide-dismutase (SOD), glutathione S-transferase (GST) and peroxidase (POD) family has been reported in salt treated pistachio (Jazi et al., 2017). The upregulation in abundance of [Cu-Zn] SOD indicates its role in conferring salt tolerance to mango genotype Turpentine. Similarly PODs and GSTs have previously been reported to increase in response to salt stress to help plant cope with oxidative damage (Jiang et al., 2007; Cheng et al., 2009). Thus, it could be suggested that anti-oxidant enzymatic machinery protects the salt stressed Turpentine from oxidative stress. Further, Late embryogenesis abundant (LEA) proteins are found to be involved in protecting plants against desiccation induced damage, maintaining ion homeostasis, protein folding and acting as chaperone proteins to protect cells against membrane damage (Grelet et al., 2005; Umezawa et al., 2006). Late embryogenesis abundant protein D was found to be upregulated in salt stressed Turpentine suggesting that it might be implicated in conferring tolerance to osmotic stress caused by salinity stress in this genotype. Among the other stress-inducible transcripts which were upregulated in Turpentine were SKP1-like protein 1 and SAL1 phosphatase. SKP1 (S-phase kinase-associated protein1) play major role in plant biology and are known to be up-regulated in response to abiotic stress (Rao et al., 2018).

### 4.11 qRT-PCR analysis

In order to validate the RNA-Seq data, qRT PCR analysis was done using three stress responsive genes viz., Late embryogenesis abundant (LEA) protein involved in osmo-protection by preventing desiccation as a result of osmotic stress caused by salt stress, Auxin response factor 2 (ARF2), involved in the transcriptional activation or repression of auxin-responsive genes and Calcium-dependent protein kinase (CDPK), involved in salt stress signalling to perform qRT-PCR analysis in the leaf samples of mango genotypes Turpentine (Salt tolerant) and Mylepelian (salt susceptible) 5, 10 and 15 days after salt treatment (**Figures 6A–C**). Contrasting expression pattern of ARF2, LEA and CDPK genes were observed between Turpentine and Mylepelian at varying salt level. A strong positive correlation between qRT-PCR results and RNA-Seq data (R2 = 0.7939) indicated the reliability of the RNA-Seq expression profile in the present study.

## 5 Conclusion

This study is the first attempt at whole transcriptome sequencing of polyembryonic mango genotypes under salinity stress which are the potential candidates for rootstock breeding in mango. The study revealed differential expression of genes under different treatment levels in salt tolerant and susceptible mango genotypes including transcription factors (bZIP, NAC, bHLH), genes involved in signal transduction (CDPK), phytohormone biosynthesis and signalling, carbohydrate and energy metabolism, calcium signalling pathway, protein kinases and stress and defence. A strong positive correlation between qRT-PCR results and RNA-Seq data (R2 = 0.7939) indicated the reliability of the RNA-Seq expression profile in the present study. Being the first report on transcriptome sequencing of mango under salinity stress, this study will help in understanding the possible molecular mechanism underlying salt tolerance in mango. The genes identified could prove to be a potential source for development of SSR markers which can serve as valuable baseline information to generate new targets for mango breeding for salt tolerance.

## Data availability statement

 The data presented in the study are deposited in the "National Library of Medicine, National Center for Biotechnology Information" repository, accession number "PRJNA930384” and "PRJNA948665" direct link/URL to this data is available at https://www.ncbi.nlm.nih.gov/sra/PRJNA930384 and https://dataview.ncbi.nlm.nih.gov/object/PRJNA948665.

## Author contributions

NP, MD, MS and KR conceived and designed the experiment. NP, HK performed the experiment. NP, KR, HK analyzed data and wrote the manuscript. VH, SA, MK and MI revised the manuscript. All authors contributed to the article and approved the submitted version.
