# Supplementary material for: Comparative transcriptome analysis provides novel insights into molecular response of salt-tolerant and sensitive polyembryonic mango genotypes to salinity stress at seedling stage
Source: Front Plant Sci. 2023 Apr 12;14:1152485. doi: 10.3389/fpls.2023.1152485 (PMC10141464; doi:10.3389/fpls.2023.1152485)
Supplement: Supplementary file 1 [file Table_1.docx]

**Comparative transcriptome analysis provides novel insights into molecular response of salt-tolerant and sensitive polyembryonic mango genotypes to salinity stress at seedling stage**

**Journal: Plant Molecular Biology**

**Nusrat Perveen^a^, M.R. Dinesh^a^, M. Sankaran^a^, K.V. Ravishankar^b*^, Hara Gopal Krishnajee^b^, Vageeshbabu S. Hanur^b^**

**^a^Division of Fruit Crops, ^b^Division of Biotechnology**

**ICAR-Indian Institute of Horticultural Crops,**

**Hesaraghatta Lakepost, Bengaluru-560089, Karnataka**

***Corresponding author: K.V. Ravishankar,** Principal Scientist

[kv_ravishankar@yahoo.co.in](mailto:kv_ravishankar@yahoo.co.in)

**Supplementary Table 1. Methods and softwares used for bioinformatics analysis**

| **Analysis** | **Software** | **Version** | **Parameter** | **Remark** |
| --- | --- | --- | --- | --- |
| Assembly | Trinity | 2.6.6 | minKmerCov=3 |  |
|  |  |  | min_glue=4 |  |
|  | Corset | 4.6 | -f ture, Default, -m 10 | remove redundancy |
|  | BUSCO | 3.0.2 | -m tran |  |
| Gene Functional Annotation | Diamond | 0.8.22 | e-value = 1e-5 | NR, KOG/COG, Swiss-Prot |
|  | Diamond, KAAS | 0.8.22 | e-value = 1e-5 | KEGG Annotation |
|  | NCBI blast | 2.9.0 | e-value = 1e-5 | NT Annotation |
|  | hmmscan | HMMER 3.1 | e-value = 0.01 | Pfam Annotation |
|  | blast2go | b2g4pipe_v2.5 | e-value = 1e-6 | GO Annotation |
| Mapping and Quantification | RSEM | 1.2.28 | --estimate-rspd - mismatch-rate 0.3 | mapping to Corset filtered transcriptome |
| Mutation | SAMtools/BCFtools | 1.9 | bcftools view varFilter -Q 20 -d 1 -D 100 | |
| SSR Analysis | MISA, primer3 | primer3-2.3.4 | SSR: 1-10 2-6 3-5 4-5 5- 5 6-5 | Misa detect SSR, primer3 Primer Design |
| Differential Expression Analysis | DESeq2 | 1.26.0 | padj< 0.05 padj | For sample with bio-replicate using DESeq2, samples without bio-replicate using EdgeR. |
|  | edgeR | 3.28.0 | padj< 0.005 & \|log2(foldchange)\| > 1 |  |
| GO Enrichment | GOSeq, topGO | 1.32.0, 2.32.0 | Corrected P-Value < 0.05 |  |
| KEGG Enrichment | KOBAS | v3.0 | Corrected P-Value < 0.05 |  |
| Protein-Protein Interaction Analysis | NCBI blast 29.0 | v2.2.28+ | e-value = 1e-10 | Using blast, String database |
