# Supplementary material for: Comparative transcriptome analysis provides novel insights into molecular response of salt-tolerant and sensitive polyembryonic mango genotypes to salinity stress at seedling stage
Source: Front Plant Sci. 2023 Apr 12;14:1152485. doi: 10.3389/fpls.2023.1152485 (PMC10141464; doi:10.3389/fpls.2023.1152485)
Supplement: Supplementary file 2 [file Table_2.docx]

**Comparative transcriptome analysis provides novel insights into molecular response of salt-tolerant and sensitive polyembryonic mango genotypes to salinity stress at seedling stage**

**Journal: Plant Molecular Biology**

**Nusrat Perveen^a^, M.R. Dinesh^a^, M. Sankaran^a^, K.V. Ravishankar^b*^, Hara Gopal Krishnajee^b^, Vageeshbabu S. Hanur^b^**

**^a^Division of Fruit Crops, ^b^Division of Biotechnology**

**ICAR-Indian Institute of Horticultural Crops,**

**Hesaraghatta Lakepost, Bengaluru-560089, Karnataka**

***Corresponding author: K.V. Ravishankar,** Principal Scientist

[kv_ravishankar@yahoo.co.in](mailto:kv_ravishankar@yahoo.co.in)

**Supplementary Table 2. List of primers used in qRT-PCR analysis**

| **Gene** | **Forward Primer** | **Reverse Primer** |
| --- | --- | --- |
| Actin | 5’ AGCGAGTCTTCATAGGGCGATTGT 3’ | 5’ TAGCTCTGGGTTCGAGTGGCATTT 3’ |
| Cluster-6633.0 | 5’-TGCACCCGACAATACATCAG-3’ | 5’-ACAGGCAGCAGTGTGATAAG-3’ |
| Cluster-5747.5087 | 5’-CTCCTGACTCTTCGATGCTTAC-3’ | 5’-GACTCCCACTGACAAGTTCAA-3’ |
| Cluster-5747.18094 | 5’-GTGCCTTCTCTGCTTCTCTTT-3’ | 5’-CGCCTGAGAACAAGCTACATTA-3’ |
