# Supplementary material for: Comparative transcriptome analysis provides novel insights into molecular response of salt-tolerant and sensitive polyembryonic mango genotypes to salinity stress at seedling stage
Source: Front Plant Sci. 2023 Apr 12;14:1152485. doi: 10.3389/fpls.2023.1152485 (PMC10141464; doi:10.3389/fpls.2023.1152485)
Supplement: Supplementary file 3 [file Table_3.docx]

**Comparative transcriptome analysis provides novel insights into molecular response of salt-tolerant and sensitive polyembryonic mango genotypes to salinity stress at seedling stage**

**Journal: Plant Molecular Biology**

**Nusrat Perveen^a^, M.R. Dinesh^a^, M. Sankaran^a^, K.V. Ravishankar^b*^, Hara Gopal Krishnajee^b^, Vageeshbabu S. Hanur^b^**

**^a^Division of Fruit Crops, ^b^Division of Biotechnology**

**ICAR-Indian Institute of Horticultural Crops,**

**Hesaraghatta Lakepost, Bengaluru-560089, Karnataka**

***Corresponding author: K.V. Ravishankar,** Principal Scientist

[kv_ravishankar@yahoo.co.in](mailto:kv_ravishankar@yahoo.co.in)

**Supplementary Table 3. qRT-PCR reaction conditions**

| **Sl. No.** | **Steps** | **Primers** | | **Cycles** |
| --- | --- | --- | --- | --- |
|  |  | **Temperature (°C)** | **Time** |  |
| 1 | Initial denaturation | 94 | 3 min | 35 cycles |
| 2 | Denaturation | 94 | 30 sec |  |
| 3 | Annealing | 60 for internal control Actin; 52 for primers for LEA, ARF2 and CDPK | 30 sec |  |
| 4 | Primer extension | 72 | 1in |  |
| 5 | Melt curve | Set to default |  |  |
