# Supplementary material for: Comparative transcriptome analysis provides novel insights into molecular response of salt-tolerant and sensitive polyembryonic mango genotypes to salinity stress at seedling stage
Source: Front Plant Sci. 2023 Apr 12;14:1152485. doi: 10.3389/fpls.2023.1152485 (PMC10141464; doi:10.3389/fpls.2023.1152485)
Supplement: Supplementary file 4 [file Table_4.docx]

**Comparative transcriptome analysis provides novel insights into molecular response of salt-tolerant and sensitive polyembryonic mango genotypes to salinity stress at seedling stage**

**Journal: Plant Molecular Biology**

**Nusrat Perveen^a^, M.R. Dinesh^a^, M. Sankaran^a^, K.V. Ravishankar^b*^, Hara Gopal Krishnajee^b^, Vageeshbabu S. Hanur^b^**

**^a^Division of Fruit Crops, ^b^Division of Biotechnology**

**ICAR-Indian Institute of Horticultural Crops,**

**Hesaraghatta Lakepost, Bengaluru-560089, Karnataka**

***Corresponding author: K.V. Ravishankar,** Principal Scientist

[kv_ravishankar@yahoo.co.in](mailto:kv_ravishankar@yahoo.co.in)

**Supplementary Table 4. Data quality control summary**

| **Sample** | **Raw reads** | **Clean reads** | **Raw bases** | **Clean bases** | **Q20(%)** | **Q30(%)** | **GC(%)** |
| --- | --- | --- | --- | --- | --- | --- | --- |
| **MT** | 4154846 | 2795088 | 1.2 | 0.8 | 82.91 | 75.53 | 49.86 |
| **MC** | 19445963 | 17535948 | 5.8 | 5.3 | 93.95 | 89.70 | 47.31 |
| **TT** | 8419934 | 7813704 | 2.5 | 2.3 | 90.47 | 85.42 | 46.59 |
| **TC** | 6433093 | 5544894 | 1.9 | 1.7 | 88.59 | 82.90 | 47.42 |
