# Supplementary material for: Comparative transcriptome analysis provides novel insights into molecular response of salt-tolerant and sensitive polyembryonic mango genotypes to salinity stress at seedling stage
Source: Front Plant Sci. 2023 Apr 12;14:1152485. doi: 10.3389/fpls.2023.1152485 (PMC10141464; doi:10.3389/fpls.2023.1152485)
Supplement: Supplementary file 5 [file Table_5.docx]

**Comparative transcriptome analysis provides novel insights into molecular response of salt-tolerant and sensitive polyembryonic mango genotypes to salinity stress at seedling stage**

**Journal: Plant Molecular Biology**

**Nusrat Perveen^a^, M.R. Dinesh^a^, M. Sankaran^a^, K.V. Ravishankar^b*^, Hara Gopal Krishnajee^b^, Vageeshbabu S. Hanur^b^**

**^a^Division of Fruit Crops, ^b^Division of Biotechnology**

**ICAR-Indian Institute of Horticultural Crops,**

**Hesaraghatta Lakepost, Bengaluru-560089, Karnataka**

***Corresponding author: K.V. Ravishankar,** Principal Scientist

[kv_ravishankar@yahoo.co.in](mailto:kv_ravishankar@yahoo.co.in)

**Supplementary Table 5. Overview of the Number of Transcripts and Unigenes in Different Length Intervals**

| **Transcript Length Interval** | **200bp-500bp** | **500bp-1kbp** | **1k-2kbp** | **>2kbp** | **Total** |
| --- | --- | --- | --- | --- | --- |
| **Number of Transcripts** | 5851 | 12260 | 16118 | 7157 | 41386 |
| **Number of Unigenes** | 5850 | 12260 | 16118 | 7157 | 41385 |
